# Role of Endophytic Fungi in the Biosynthesis of Metal Nanoparticles and Their Potential as Nanomedicines

**DOI:** 10.3390/jfb16040129

**Published:** 2025-04-03

**Authors:** Hanadi Sawalha, Simon E. Moulton, Andreas Winkel, Meike Stiesch, Bita Zaferanloo

**Affiliations:** 1 Department of Chemistry and Biotechnology, School of Science, Computing and Engineering Technologies, Swinburne University of Technology, Hawthorn, VIC 3122, Australia; 2 School of Engineering, Swinburne University of Technology, Melbourne, VIC 3122, Australia; smoulton@swin.edu.au; 3 Aikenhead Centre for Medical Discovery, St Vincent’s Hospital Melbourne, Melbourne, VIC 3065, Australia; 4 Iverson Health Innovation Research Institute, Swinburne University of Technology, Hawthorn, VIC 3122, Australia; 5 Department of Prosthetic Dentistry and Biomedical Materials Science, Hannover Medical School, 30625 Hannover, Germany; winkel.andreas@mh-hannover.de (A.W.); stiesch.meike@mh-hannover.de (M.S.); 6 Lower Saxony Center for Biomedical Engineering, Implant Research and Development (NIFE), 30625 Hannover, Germany

**Keywords:** endophytic fungi, biosynthesis, MNPs, anticancer, antimicrobials

## Abstract

Metal nanoparticles (MNPs) produced through biosynthesis approaches have shown favourable physical, chemical, and antimicrobial characteristics. The significance of biological agents in the synthesis of MNPs has been acknowledged as a promising alternative to conventional approaches such as physical and chemical methods, which are confronted with certain challenges. To meet these challenges, the use of endophytic fungi as nano-factories for the synthesis of MNPs has become increasingly popular worldwide in recent times. This review provides an overview of the synthesis of MNPs using endophytic fungi, the mechanisms involved, and their important biomedical applications. A special focus on different biomedical applications of MNPs mediated endophytic fungi involved their antibacterial, antifungal, antiviral, and anticancer applications and their potential as drug delivery agents. Furthermore, this review highlights the significance of the use of endophytic fungi for the green synthesis of MNPs and discusses the benefits, challenges, and prospects in this field.

## 1. Introduction

Nanotechnology has significantly advanced in several fields, including synthesising nanoscale materials and exploring the application of their unique physicochemical and optoelectronic properties [1]. Recent advancements in understanding self-assembly mechanisms have resulted in the emergence of novel methodologies for nanoparticle arrangement into predetermined superstructures [2]. Consequently, nanotechnology is anticipated to assume a progressively significant position in numerous pivotal technologies in the next era [3]. Arising from this, there is a growing interest in establishing methodologies for the synthesis and assembly of nanoparticles (NPs) that are clean, non-toxic, and environmentally sustainable, sometimes referred to as “green chemistry” [4]. As a result, researchers have begun to extensively explore biological systems as a source of inspiration for clean synthesis methods [5].

Over the past decade, scientists have focused significantly on the use of metal nanoparticles (MNPs) in biological and biomedical fields owing to their outstanding optical properties, controlled size and form, and relatively simple synthesis [6]. The study of MNPs, with their novel characteristics and wide range of potential uses, has recently become an exciting topic of nanotechnology study [7]. The optical, electrical, and catalytic properties of these nanoscale structures with a typical size ranging from 1 to 100 nanometres are significantly different from those of their bulk counterparts [8,9]. For example, MNPs can be used as biopolymers [10,11], hydrogel MNPs [12], and composite scaffolds [13]. Furthermore, remarkable developments are being made in nanobiotechnology because of the size and shape-dependent characteristics of MNPs, with these properties having a significant influence on several aspects of human life [14]. These NPs have promising applications in various areas, including the environment, agriculture, electronics, and medicine [15].

Different physical and chemical procedures have been employed for NP synthesis, namely chemical reduction methods, mechanical milling, polyol-assisted, thermal decomposition, and laser ablations [16]. In physical processes, the use of radiation, pressure, and high temperature may result in serious damage to living things [17]. Chemical methods are used to efficiently produce uniform NPs within several hours while maintaining control over their size and distribution [18]. However, chemical processes use harmful substances, require a lot of energy, and generate hazardous waste that poses a significant environmental risk when exploited in environmental applications [19]. Likewise, physicochemical methods of synthesising NPs result in a low yield rate, have high costs, and require a prolonged procedure that releases harmful substances into the atmosphere [20].

In contrast, biological techniques are quick and cost-effective resulting in the production of non-toxic, environmentally friendly NPs [21]. Furthermore, biogenic reduction in an aqueous solution leads to the formation of stable, uniform, and polydisperse MNPs [22]. Plant extracts, bacteria, yeasts, actinomycetes, and fungi are known to manufacture NPs [20]. The production of MNPs using microorganisms can be primarily categorised into biological synthesis and biotemplated production approaches. The biological synthesis approach primarily depends on the metabolic processes of natural organisms, leading to the random distribution of nanoparticles either within or outside the microbial cells. Furthermore, the biotemplated forming approach uses microorganisms’ substructure or whole morphology as binding sites or templates to create ordered nanoparticle aggregation or regular functioning microparticles by various chemical or physical deposition procedures [23].

Fungi are considered the most reliable source for stable metal nanoparticle synthesis [20], and are preferred over other microorganisms such as bacteria and viruses owing to their easy scaling up of further processing, affordability, and mycelia [24]. They secrete a diverse range of enzymes and proteins compared to other microorganisms like bacteria, enabling them to efficiently generate metal nanoparticles on a large scale in a short period [25].

Endophytic fungi are microorganisms that reside within healthy plant tissues without creating any infectious symptoms [26] and can be found on all plants in nature [27]. Endophytic fungi produce numerous enzymes, secondary metabolites, alkaloids, and novel compounds with significant medical and industrial applications [28]. The fungi are also ideal for producing MNPs due to their ability to generate substantial MNP biomass, providing a high yield of NPs [29].

In the last few years, MNPs synthesised by endophytic microbes have shown tremendous promise in the biomedical arena, bringing in novel possibilities for diagnostics, therapies, and bioimaging [30]. The current review aims to highlight the current scenario of the biosynthesis of MNPs from endophytic fungi, as an eco-friendly technique for obtaining these NPs. For this purpose, an insight into endophytic fungi as nano-factories is reported along with the synthesis, characterisation, and various biomedical applications of MNP-mediated endophytic fungi, as well as research limitations and challenges.

## 2. Overview of Fungal Endophytes

**Figure 1:**
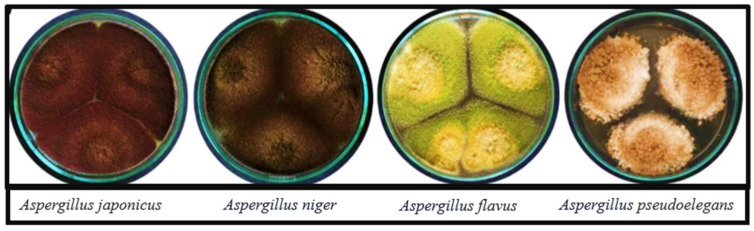
Morphological representation of different endophytic fungi *Aspergillus* species. This figure has been adopted from Atallah et al. [37] with slight modifications.

Plants carry a tremendous range of microorganisms living within their tissues [31]. Endophytes are microorganisms that reside all or part of their life cycle inside the plant tissues, where they typically do not cause any harm or disease symptoms to the host plant [32]. A plant can be home to several endophytic species, which remain in one place and protect their tissues from disease [33] or completely spread within the herbaceous plants [34]. Examples of endophytic fungi are shown in Figure 1. The term “fungal endophytes” refers to a wide variety of microorganisms that establish and maintain mutualistic or symbiotic interactions with their host plant while living asymptomatically within the plant’s tissues [35]. The symbiotic interactions between plants and endophytes are varied and encompass both wild and cultivated plant species [36].

Various types of fungi can become endophytes, and they fall into many different taxonomic categories [38]. Researchers are only at the tip of the endophytic iceberg, with only a small percentage of the estimated 1 million endophytic species having been discovered among 300,000 plant species that have been isolated and studied for their functions [39]. Endophytic fungal communities are as varied as the hosts they inhabit, with the majority of endophytic fungi classified under the division Ascomycota [40,41], according to recent reports. The most prevalent ascomycetes are *Sordariomycetes*, *Dothideomycetes*, *Eurotiomycetes* and *Leotiomycetes* [42]. Endophytic fungi generally exhibit significant diversity, including over 70 distinct fungal orders [43]. The most renowned fungal orders include Pleosporales, Hypocreales, *Xylariales*, *Eurotiales*, *Helotiales*, and *Botryosphaeriales* [44]. *Pleosporales* have been classified as the largest order within the Dothideomycetes, which constantly expands with the discovery of new families, genera, and species [45]. Regarding the most prominent genera among the endophytes, *Penicillium* is the species with the highest number of genus species, followed by *Alternaria*, *Fusarium*, *Colletotrichum*, *Aspergillus*, and *Xylaria* in subsequent order [46].

Endophytic fungi are an area of great interest in different biomedical fields [47]. They can release secondary metabolites that are both beneficial and intriguing due to their capacity to tolerate and bioaccumulate metals [48]. Several important groups of secondary metabolites, including alkaloids, terpenoids, phenols, enzymes, and organic acids exhibit a wide range of biomedical activities [49]. These activities include antioxidants, antibiotics, antimicrobials, antidiabetic, antiarthritic, anti-inflammatory, and immunosuppressant activities, which could be used to solve the challenge of finding novel therapies to cure human diseases [50]. Furthermore, the aforementioned biomolecules and enzymes are commonly responsible for the synthesis of stabilised and non-agglomerated MNPs. Various endophytic fungi have shown the ability to synthesise MNPs for biomedical applications such as *Aspergillus terreus* [51], *Penicillium oxalicum* [51], *Penicillium cinnamopurpureum* [52], *Puccinia helianthin* [53], *Penicillium* sp. [54], Lasiodiplodia pseudotheobromae [55], and *Periconium* sp. [56]. Understanding endophytic fungi has the potential to contribute to the development of sustainable products that are in line with the goals of sustainability. An important use is the adaptation of endophytic fungi for the biological synthesis of MNPs owing to their scalability and cost-effectiveness [57].

## 3. Synthesis of Metal Nanoparticles by Endophytic Fungi

The fabrication of MNPs can be achieved using two different approaches, namely the top-down method and the bottom-up method [58,59]. In addition, three separate approaches, mainly physical, chemical, and biological procedures, are employed for fabricating MNPs [60].

**Figure 2:**
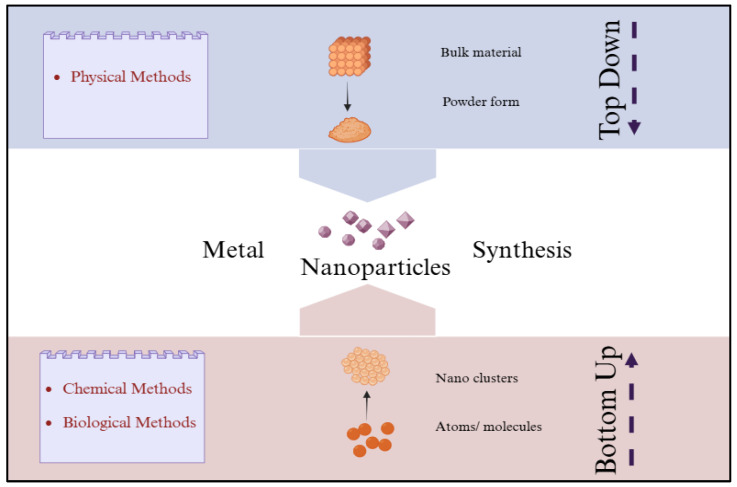
Bottom-up and top-down approaches to synthesis of NPs including chemical, biological, and physical methods. Created with BioRender.com “https://app.biorender.com/ (accessed on 18 March 2025)”.

Synthesis of MNPs via physical processes is an example of a top-down approach, whereas methods involving chemicals and biology are examples of bottom-up approaches [61], as shown in Figure 2. Physical processes such as evaporation–condensation, electrolysis, sputter deposition, plasma arcing, laser ablation, and high-energy ball milling are commonly employed for the synthesis of MNPs [62]. However, physical processes have drawbacks of generating toxic waste, expensive, low production, and high energy requirements [63]. In contrast, the conventional and most frequently used chemical processes for synthesising MNPs involve chemical reduction, thermal decomposition, and micro-emulsion [64]. One of the most popular means for synthesising MNPs chemically is the chemical reduction of MNPs from their respective metal salt predecessors [65]. This process requires little in the way of operating or equipment requirements, with the chemical procedures being cost-effective for large-scale MNP production [65]. However, the use of toxic chemicals and the generation of dangerous by-products result in environmental damage, therefore restricting its therapeutic and biological applications [66]. Therefore, there is a growing demand for reliable, high-yield, and environmentally sustainable approaches for MNP production that can substitute for traditional methods.

**Figure 3:**
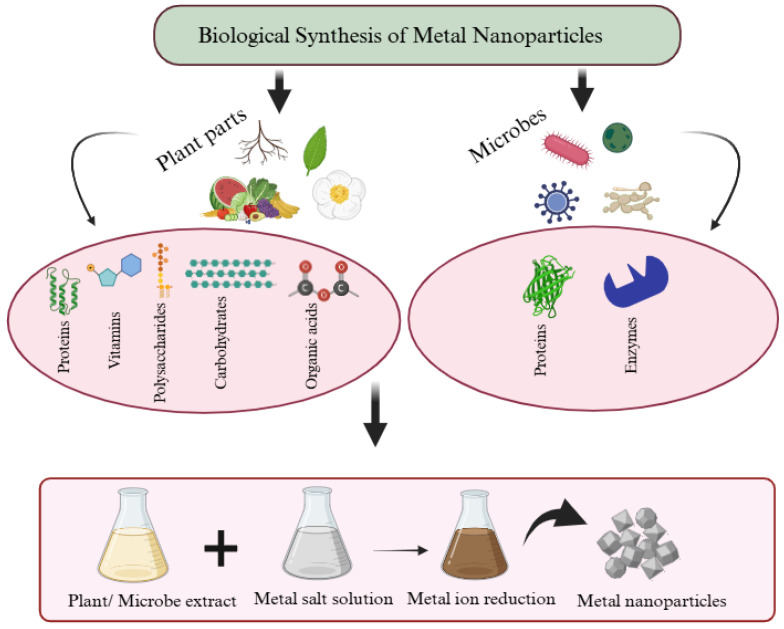
A generalised approach to the biosynthesis of MNPs mainly using plants and microbes as biological entities. Created with BioRender.com. “https://app.biorender.com/ (accessed on 18 March 2025)”.

Biological synthesis approaches offer a compelling alternative to physicochemical synthesis approaches [67]. The biosynthesis of MNPs has primarily relied on the utilisation of plants and microorganisms [67]. These procedures involve the sustainable use of plants, microorganisms, or their derived components as reducing and stabilising agents, thereby eliminating the need for supplementary chemicals [68]. Plant parts, such as seeds, roots, leaves, stems, and fruits, are commonly used for the synthesis of different MNPs [67], whilst plant extracts can generate MNPs with particular shapes, sizes, and compositions [69]. In addition, plant extracts contain an array of phytochemicals that may be used as natural stabilisers and/or reducing agents for the formation of MNPs [70]. These bioactive compounds include proteins, organic acids, polysaccharides, amino acids and phytochemicals such as flavonoids, alkaloids, polyphenols, terpenoids, and alcoholic substances [70]. Indeed, both plants and microorganisms possess distinct advantages, that result in the process of plant-based synthesis typically exhibiting simplicity and quickness [71]. However, the main disadvantage of using plant-based products is that it can result in the production of MNPs with various shapes and sizes resulting in polydispersity [72], as illustrated in Figure 3. Akinfenwa, Abdul, Docrat, Marnewick, Luckay, and Hussein [73] synthesised AgNPs and AuNPs using Aspalathus linearis extract with the results showing diverse shapes and sizes of AgNPs and AuNPs ranging from 1.6 to 6.7 nm and from 7.5 to 12.5 nm, respectively. Cu_2_O synthesised using *Plukenetia volubilis* L. extract exhibited a spherical and semi-crystalline shape with an average size of 6–10 nm [74]. Niculescu, Chircov, and Grumezescu [75] reported that plants have a wide variety of active compounds including glycosides, flavonoids, and terpenoids that have the potential to stabilise MNPs, thereby contributing to this polydispersity. Thus, there is a need for another green and safe MNP synthesis. Microbes can act as cost-effective and secure means for producing MNPs, notably silver, nickel, gold, titanium, copper, palladium, and zinc [76].

There is an emerging shift towards the biosynthesis of NPs using eukaryotic organisms such as fungi. The size, shape, and surface morphology of the NPs are significantly controlled by the secreted enzymes and biomolecules released by fungal endophytes [77]. The ability to control the shape of NPs is essential for improving their functionality and applications [78]. Several studies have shown that certain fungi secrete chemicals extracellularly such as proteins and enzymes that serve as agents to ensure their survival in the face of harmful environmental challenges, including poisonous substances (such as metal ions), and temperature changes [79]. These compounds possess the essential biological components to reduce metal ions to MNPs, stabilise them, and facilitate an efficient synthesis process [79]. Through the biosynthesis of MNPs using fungi, the fungi mycelium is kept in a metal salt solution [80], allowing the fungus to generate the necessary metabolites and enzymes for its endurance [81]. In this process, toxic metal ions change into non-toxic MNPs through the catalytic activity of the extracellular enzyme and metabolites produced by the fungi [76]. This is attributed to biological synthesis employing moderate reaction conditions and biological reducing agents, hence minimising dangerous chemicals and decreasing toxicity in the produced NPs [82].

**Figure 4:**
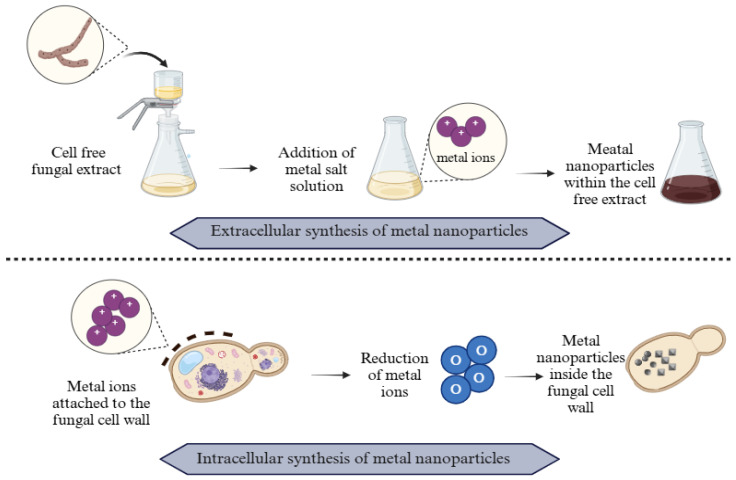
Schematic representation for the intracellular and extracellular synthesis of MNPs from endophytic fungi. Created with BioRender.com “https://app.biorender.com/ (accessed on 18 March 2025)”.

As shown in Figure 4, fungi can be exploited for MNP fabrication through both intracellular and extracellular methods. During intracellular production, heavy metals bind to the fungal cell wall through proteins or enzymes, leading to electrostatic interactions [83]. As a result, metal ions are reduced and electrons are transferred [83]. During the extracellular production of MNPs, metal ions interact with enzymes, particularly reductase, leading to the formation of NPs in the solution [84]. It is suggested that the reductases facilitate the reduction of ions into their corresponding MNPs and are essential for the extracellular biosynthesis of MNPs [85]. Additionally, the size and morphology of synthesised NPs can be controlled by adjusting reaction parameters including pH, temperature, metal precursor concentration, and reaction time [86]. A study found that optimised parameters of biosynthesis of polydispersed-AgNPs were 10 mL of fungal extract, precursor concentration (1 mM), the ratio between culture extract–precursor concentration (10:90 mL), temperature (37 C), pH 9, and 24 h of incubation time resulted in stable and polydispersed AgNPs [87]. Another study, by Dinesh et al. [88], assessed the influence of AgNO_3_ concentration, pH, and reaction time. The study concluded that 5 mM AgNO_3_, pH 9, for 3 h of reaction time were the optimum conditions for the production of AgNPs mediated by *Simpicillium lanosoniveum*.

**Figure 5:**
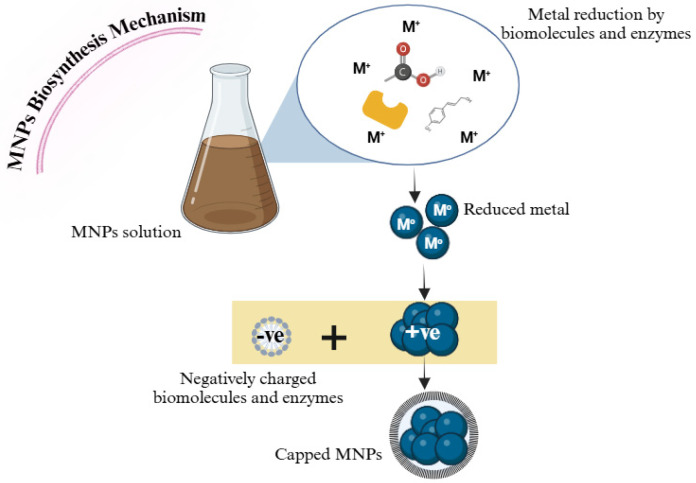
Schematic diagram of the mechanism by which endophytic fungi act as nano-factories to synthesise MNPs. The metal reduction is attributed to biomolecules and enzymes in the endophytic fungal extract, which are bio-capping agents of produced MNPs. Created with BioRender.com “https://app.biorender.com/ (accessed on 18 March 2025)”.

Additionally, biological methods exploit safer solvents and reaction conditions, employing simple procedures that can be duplicated and scaled up without intricate changes or extra equipment. These methods also involve using low-cost raw materials and equipment that require less expensive maintenance and operation. Moreover, single-step approaches combine various stages of a process into a single step, typically by employing advanced chemistry that enables simultaneous or sequential reactions within one step. The biosynthesis of MNPs by fungi is initiated by adding a metal salt precursor to the fungal extract [89]. The existence of various substances such as reducing sugars, alkaloids, polyphenols, flavonoids, and proteins in the fungal extract serve as reducing and capping agents during the synthesis of MNPs [85]. Reducing sugars contain free aldehyde or ketone functional groups that are capable of donating electrons [90]. These electrons act as reducing agents, converting metal ions (such as Ag^+^ and Au^3+^) into their corresponding zero-valent metal forms (Ag^0^ and Au^0^) [90]. For instance, glucose can reduce Ag^+^ into AgNPs [91] with polyphenols possessing many hydroxyl groups that can function as electron donors [11,91]. They catalyse the reduction of metal ions via donating electrons, resulting in the production of MNPs [92]. Tannins and catechins are examples of polyphenolic compounds that are included in these reactions [93]. Flavonoids have several phenolic structures that can donate electrons [94] and have the ability to reduce metal ions by utilising their hydroxyl groups, which leads to the production of MNPs [94]. Polyphenols and flavonoids can also act as capping agents by binding to the surface of MNPs through their hydroxyl groups [69] and have a stabilising impact, preventing the MNPs from aggregating [95]. Alkaloids are chemicals that include nitrogen and can engage in redox reactions [96]. Due to their molecular structure, they can transfer electrons to metal ions, resulting in the formation of MNPs [96]. Proteins include functional groups such as thiols, amins, and carboxyl that can attach to metal surfaces [97] and following the reduction of metal ions, provide stabilisation through the prevention of aggregation [95]. The steric stabilisation of MNPs is attributed to the protein’s large size and complicated structure, which forms an outer layer around MNPs [98]. Other organic chemicals found in fungal extracts, such as carboxyl, hydroxyl, and amino groups, can act as capping agents by binding to the surfaces of MNPs [99]. As a result, this binding provides electrostatic and stabilisation to the biosynthesised MNPs, as depicted in Figure 5.

The confirmation of the reduction of a metal salt precursor to its subsequent MNPs can be initially achieved by observing the colour change of the final solution [100]. The colour change occurs due to the production of MNPs, which frequently exhibit unique optical characteristics that differ from those of the original metal salt precursor [7]. Various MNPs, particularly those composed of noble metals such as gold (Au) and silver (Ag), demonstrate a phenomenon called localised surface plasmon resonance (LSPR) [101]. LSPR is the phenomenon where conduction electrons on the surface of a nanoparticle vibrate in response to incident light of certain wavelengths [102]. The oscillations cause significant absorption and scattering of light, leading to the emergence of distinct colours [103]. For example, the AgNO_3_ solution is colourless and, once reduced to form the AgNPs, typically displays a yellowish-brown colour owing to the LSPR [104]. Bagur, Medidi, Somu, Choudhury, Karua, Guttula, Melappa, and Poojari [54] indicated the presence of AgNPs after the change of a light-yellow fungal extract to a brown colour. Another example is the chloroauric acid (HauCl_4_) solution, which is normally pale yellow and, after the reduction, the AuNPs represent a dark red to purple colour that varies depending on the size and shape of the NPs. The fungal biomass experienced a colour change to red after being immersed in a 1 mM HauCl_4_ solution for 48 h [105]. To conclude, this is a simple and fast way to observe the progress of MNP formation that later should be followed by monitoring using different characterisation tools.

## 4. Characterisation Techniques

Characterising MNPs is mandatory to determine their functional, morphological and structural properties [106]. Morphological characterisation gives beneficial information about their surface morphology, size, and shape [107]. Nanoparticle size distribution, morphology, and surface characteristics can be observed in high resolution using techniques like transmission electron microscopy (TEM) and scanning electron microscopy (SEM) [108]. In addition, the three-dimensional topographical data can be detected using atomic force microscopy (AFM) [109]. TEM is a widely recognised tool for the characterisation of MNPs which utilises an electron beam to interact with a specimen, resulting in an image on a photographic film [110]. TEM is renowned for its ability to accurately identify and measure the chemical and electronic composition of NPs [111]. TEM possesses certain advantages in comparison to SEM, such as enhanced spatial resolution capabilities and presents supplementary analytical measurements for NPs such as electron diffraction to analyse the crystal structure of NPs, and electron tomography to analyse the morphology of NPs [112].

Studies conducted on the synthesis of MNPs using endophytic fungi have been performed with different characterisation tools to investigate the morphological and chemical properties of MNPs. For example, TEM and SEM were used to investigate the size and shape of the synthesised AgNPs [54]. In this study, the average size of the AgNPs was determined to be approximately 25–35 nm, exhibiting a nearly spherical shape. The UV–visible spectra of endophytic fungi-mediated AgNPs exhibited an SPR peak at 429 nm, indicating the production of AgNPs and the spherical shape of the synthesised NPs [113]. In the same study, the presence of several metabolites and proteins in the fungal filtrate, which is responsible for producing AgNPs, was also confirmed using FTIR. Various imaging and analytical techniques, including UV–vis, XRD, FTIR, TEM, and SEM, were used to analyse and describe the biosynthesised AgNPs and AuNPs using *Aspergillus* sp. and *Alternaria* sp. [114]. Another study by Munawer, Raghavendra, Ningaraju, Krishna, Ghosh, Melappa, and Pugazhendhi [115], the biosynthesised AuNPs by Cladosporium sp. were analysed using UV–vis spectroscopy, FTIR, TEM, and XRD.

Characterisation techniques are crucial for gaining valuable insights into the properties and behaviour of MNPs, guaranteeing that they fulfil the specific requirements of different biomedical applications [116]. By conducting a thorough analysis, researchers may create MNPs that are efficient and customised for certain diagnostic and therapeutic applications, thereby improving their clinical usefulness and influence [117].

## 5. Biomedical Applications

**Figure 6:**
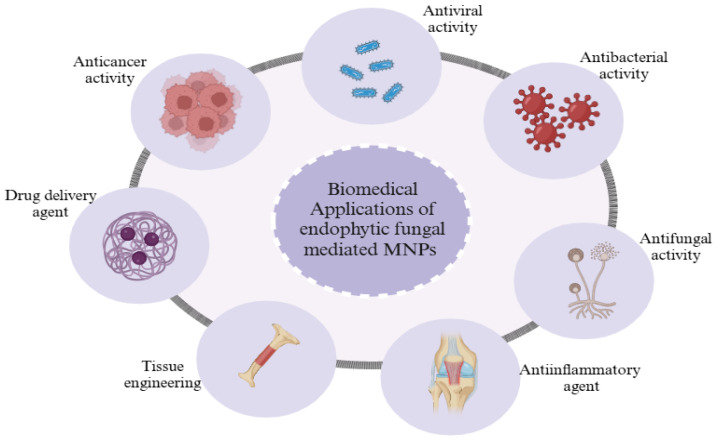
Biomedical applications of MNPs mediated by endophytic fungi. The main applications included antifungal activity, antibacterial activity, antiviral activity, anticancer activity, tissue engineering, drug delivery agents, and anti-inflammatory agents. Created with BioRender.com “https://app.biorender.com/ (accessed on 18 March 2025)”.

Nanotechnology is a game-changer in the race for novel approaches to biomedical problems [118]. In particular, MNPs have attracted much interest for their unique features and prospective uses in a wide range of biomedical fields such as drug delivery, tissue engineering [119], and tumour therapy [120], as shown in Figure 6. This approach offers promising prospects for creating cutting-edge nanomaterials with custom features for biomedical applications by capitalising on the symbiotic interaction between endophytes and plants [121]. Further, Table 1 provides a description and examples of the various biomedical applications of fungal-mediated MNPs.

### 5.1. Antibacterial Activity

**Figure 7:**
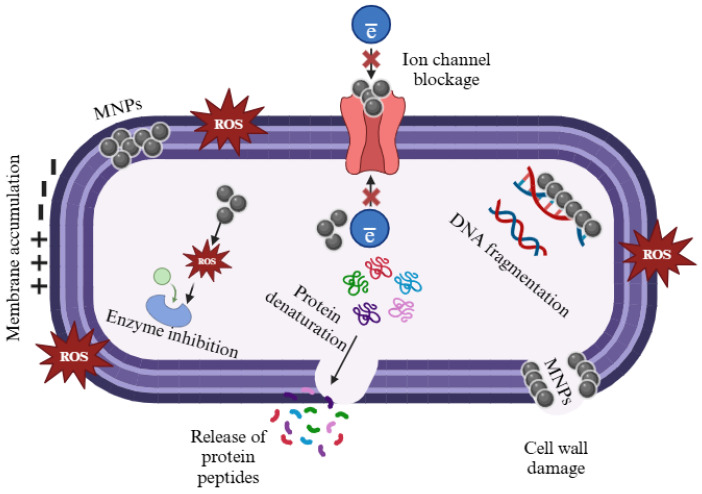
Antibacterial potential of MNPs. MNPs enter the bacterial cell through the bacterial cell wall causing cell wall damage, DNA fragmentation, enzyme inhibition, and protein denaturation by reactive oxygen species (ROS). Created with BioRender.com “https://app.biorender.com/ (accessed on 18 March 2025)”.

Generally, exposure to biosynthesised MNPs causes cytotoxicity in bacterial cells [129]. The interaction between metal ions and MNPs with the bacterial plasma membranes causes the prevention of DNA replication, disruptions in cell permeability, and cellular respiration [130]. This can be achieved by denaturing the ribosomes or binding the DNA [130], as illustrated in Figure 7. The development of drug-resistant bacterial pathogens in humans has created a pressing demand for novel and efficacious compounds that can offer assistance and relief to individuals infected with hazardous pathogens [131]. Generally, biosynthesised MNPs exhibit exciting antibacterial properties that have the potential to act as a novel alternative for the development of antibacterial drugs within the pharmaceutical industry [132]. AgNPs mediating the endophytic fungus *Penicillium polonicum* isolated from *Chetomorpha antennina* exhibited a strong antibacterial activity towards multidrug-resistant Acinetobacter baumannii [133]. Additionally, biosynthesised AgNPs produced by endophytic fungi exhibit anti-lipoxygenase, anti-dermatophytid activity, anti-inflammatory, xanthine oxidase, and tyrosine inhibitory activity [134]. AgNP-mediated *Talaromyces purpureogenus* exhibited strong antibacterial activity against different bacterial pathogens (*Staphylococcus aureus*, *Pseudomonas aeruginosa*, and *Escherichia coli*) [128]. Dinesh, Monisha, Shalini, Prathap, Poyya, Shantaram, Hampapura, Karigar, and Joshi [52] reported that AgNPs synthesised from Penicillium cinnamopurpureum possess the potential as an antibacterial agent, effectively targeting pathogenic bacteria. Feroze, Arshad, Younas, Afridi, Saqib, and Ayaz [135] found that AgNPs fabricated by Penicillium oxalicum have considerable activity against resistant bacteria, preventing infections, promoting wound healing, and reducing inflammation. Furthermore, AgNPs from Cassia fistula AgNPs showed significant antibacterial efficacy against *Staphylococcus aureus*, *Escherichia coli*, and *Klebsiella pneumoniae* bacterial strains [136]. Halkai, Mudda, Shivanna, Rathod, and Halkai [137] demonstrated that fungal-mediated AgNPs exhibited significant antibacterial activity against *Porphyromonas gingivalis*, *Enterococcus faecalis*, and *Bacillus pumilus*.

### 5.2. Antifungal Activity

Biologically derived MNPs have been studied for their antifungal activities. There have been few findings on the antiviral capabilities of fungal-mediated MNPs. Extracellularly synthesised AgNPs by *Alternaria alternata* exhibited antifungal activity against *Fusarium semitectum*, *Phoma glomerata*, *Trichoderma* sp., and *Trichoderma* sp. [138]. However, additional investigation into MNPs as antifungal medicines is crucial to finding novel compounds with reduced adverse effects. Understanding antifungal treatments is essential for effectively controlling persistent and developing fungal infections and ensuring public health protection.

### 5.3. Antiviral Activity

In general, viruses are causative agents of a diverse array of diseases such as asthma, inflammatory bowel disease, and colorectal cancer [139]. However, there is a lack in the literature on fungal-mediated MNPs and their antiviral activity. *Aspergillus ochraceus*-derived AgNPs have been investigated for their antiviral agent against M13 phage virus [140]. Antiviral activity of fungal-mediated AgNPs against herpes simplex virus and human parainfluenza virus type 3 have been studied [141]. Authors found that AgNPs can reduce the ability of viruses to infect cells, most likely by inhibiting the interaction between the virus and the cell.

### 5.4. Anticancer Activity

Cytotoxicity of biosynthesised MNPs against different cancer cell lines has been thoroughly reported [142,143]. AgNPs have demonstrated cytotoxic effects on several kinds of cancer cell lines, including HeLa, A549, MCF-7, SKOV_3_, and normal cell lines [144]. Akther, Mathipi, Kumar, Davoodbasha, and Srinivasan [122] demonstrated that AgNPs synthesised from *Botryosphaeria rhodina* have the potential to trigger apoptosis in A549 lung cancer cells, offering a new approach to treating lung cancer. A study has found that AgNPs *Penicillium oxalicum* demonstrated potent cytotoxic activity against MRC-5 cancer cells (human foetal lung fibroblast) [145]. Another study revealed that AgNPs synthesised using *Penicillium italicum* showed significant anticancer activity and cytotoxic effects against MCF-7 cells (human breast cancer) [146]. Therefore, biosynthesised AgNPs hold significant prospects for biomedical applications. AgNPs derived from *Cladosporium perangustum* exerted a notable inhibitory against MCF-7 cancer cells [127].

**Figure 8:**
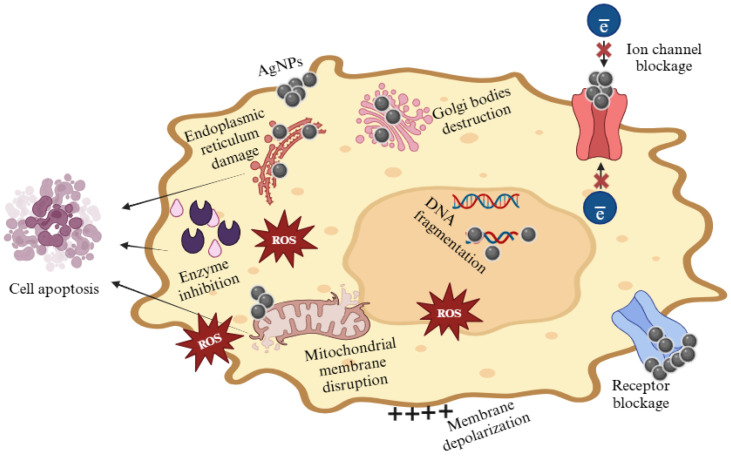
Anticancer potential of MNPs. MNPs disrupt the functioning of organelles and enzymes, especially in mitochondria, endoplasmic reticulum, and Golgi bodies, through the generation of reactive oxygen species (ROS) and inducing apoptosis. Blockage of ion channels results in the demise of malignant cells. MNPs destroy cancerous cells by degrading nucleic acids, specifically DNA. Created with BioRender.com “https://app.biorender.com/ (accessed on 18 March 2025)”.

AuNPs have also attracted significant attention from nanotechnologists as a type of MNP [147,148]. Biosynthesised AuNPs were accomplished using a suspension of mycelia of *Fusarium oxysporum* derived from *Azadirachta* indica. The synthesised AuNPs exhibited noteworthy antiproliferative properties against breast cancer and human Burkitt’s lymphoma cancer cells while demonstrating full safety towards normal human peripheral blood mononuclear cells [149]. Endophytic fungi possess significant potential for the biosynthesis of AuNPs due to their secretion of distinct metabolites and enzymes in comparison to their counterparts. This unique characteristic may facilitate the process of capping and stabilising the MNPs [150]. Fungal-mediated AuNPs had significant cytotoxic effects on MCF-7 and HeLa cancer cells [124]. The *Fusarium solani*-derived AgNPs indicated strong anticancer activities against MCF-7 and HeLa cells [124]. An endophytic fungus *Alternaria alternata* obtained from the Catharanthus roseus medicinal plant was used for the synthesis of AuNPs [151]. Kaushal [152] found that AgNPs synthesised using *Botryosphaeria rhodian*, demonstrated their cytotoxicity against lung cancer cell lines. These MNPs were used to examine the cytotoxicity against the A549 cancer cell line using the MTT assay method. As a result, fungal endophyte-mediated MNPs act as a promising tool in cancer therapy, as shown in the mechanism of anticancer potential of MNPs in Figure 8.

### 5.5. MNPs as Drug Delivery Agents

As detailed above, fungal-mediated synthesis facilitates the production of MNPs with controlled size, shape, and surface characteristics [153]. These characteristics can be effectively functionalised with specific receptors or antibodies to allow specific drug delivery [154]. Therapeutic chemicals can be directed to specific cells or tissues by attaching them to the surface of NPs, boosting therapy efficacy while minimising adverse effects [155]. Furthermore, their biocompatibility mitigates the risk of adverse effects and enhances overall safety [155].

## 6. Recommendations and Future Perspectives

Although biosynthesis of MNPs by endophytic fungi, being a green approach, has gained more attention in recent years, it is still a novel concept that is in the early stages of development. The synthesis of fungal nanoparticles shows significant potential for the future; however, several obstacles must be resolved to progress this technology. In future, it is crucial to understand the forthcoming challenges to efficiently using biosynthesised MNPs for biomedical applications.

One major issue is variability: the size and morphology of fungal NPs could vary substantially due to varying growth conditions and metal ion concentrations. This heterogeneity impacts reliability, complicating the constant production of nanoparticles with uniform characteristics. Changes resulting from several stimuli can affect the stability, toxicity, and reactivity of nanoparticles generated from endophytic fungus. These fungal nanoparticles demonstrate size-dependent characteristics; nevertheless, attaining a consistent nanoscale is difficult due to the intrinsic fluctuations and unpredictable circumstances present in biological systems during nanoparticle synthesis.

As a result, optimising the reaction conditions for NP synthesis by endophytic fungi is important. It can greatly enhance the homogeneity and stability of the MNPs, which is crucial for maintaining their consistent biological activity. Successful optimisation can also reduce undesirable variability in size and performance.

Another challenge is scalability; although the method demonstrates promise, the scaling of fungal MNP production can be intricate and expensive. Industrial production processes must be optimised, frequently requiring the management of complex and costly techniques. This scaling concern can affect the overall viability of large-scale programmes. Moreover, monitoring and regulating nanoparticle production on a larger scale becomes progressively more challenging. Factors including growth conditions, sustenance availability, and fungal metabolism may vary, resulting in inconsistencies in the final output. Addressing these challenges necessitates the development of exact methods and efficient monitoring systems to guarantee consistency in MNP production. This will facilitate more dependable and efficient production, particularly for industrial applications.

In addition, transferring fungal endophyte MNPs from laboratory settings to clinical applications involves broad preclinical and clinical investigations. These assessments encompass both in vitro and in vivo studies to evaluate their potential for inducing cytotoxicity, immunotoxicity, safety, efficacy, pharmacokinetics, and long-term impacts on biological systems. Meanwhile, it is mandatory to effectively navigate the regulatory approval process to adhere to the requisite regulations and guidelines.

Understanding the relationship between MNPs and living organisms is of the highest priority to guarantee their safety and effectiveness. Before the implementation of endophytic fungi MNPs in biomedical applications, it is necessary to conduct comprehensive assessments of their biocompatibility and toxicity.

## 7. Conclusions

The current article carefully reviewed the recent advancement in the endophytic fungi-assisted synthesis of MNPs and their biomedical applications. Endophytic fungi-mediated synthesis has great potential for synthesising various types of MNPs in a straightforward, environmentally friendly, and cost-efficient manner. This is attributed to the organic compounds secreted by endophytic fungi, which act as bio-coating materials for MNPs. By implementing established protocols and novel methodologies, it is possible to revolutionise the biosynthesis of MNPs in both laboratory and biomedical sectors. In addition, this review provides a comprehensive analysis of the benefits of the biosynthesis approach compared to traditional chemical synthesis methods, as well as highlights the future opportunities in this area. The subject of biosynthesis is recently evolving, and ongoing research focuses on the creation, characterisation, and functionalisation of MNPs. However, these processes are still in the developmental stage. To acquire adequate knowledge, it is necessary to possess comprehensive research on its fundamental concepts and engage in further research attempts. By implementing established protocols and novel methodologies, it is possible to revolutionise the synthesis of MNPs in both laboratory and commercial sectors, thereby addressing the existing challenges in this field.

## Figures and Tables

**Table 1:** Green synthesis of MNPs by different species of endophytic fungi, their size, shape, and potential activities.

Host Plant	Endophytic Fungi	MNPs	Size (nm)	Shape	Activity	References
*Centella asiatica*	*Aspergillus versicolor*	AgNPs	3–40	Spherical	Strong antimicrobial agent and effective free radical scavenging activity	[113]
*Catharanthus roseus*	*Botryosphaeria rhodia*	AgNPs	<20	Spherical	Anticancer material against A549 cancer cell line	[122]
	*Trichoderma hamatum*	AuNPs	5–30	Spherical, Pentagonal, and Hexagonal	Antimicrobial activity against bacillus subtilis, staphylococcus aureus, pseudomonas aeruginosa, and Serratia	[123]
*Chonemorpha fragrance*	*Fusarium solani*	AuNPs	~40 and 45	Spindle	Anticancer agent	[124]
*Nothapodytes foetida*	*Cochliobolus geniculatus*	ZnO	2–6	Spherical	n/a	[125]
*Tinospora cordifolia*		AgNPs	25–35	Spherical	Antibacterial, antioxidant, and anti-inflammatory activity	[54]
*Amoora rohituka*	*Penicillium oxalicum*	AgNPs	13–23	Spherical	Antiproliferative activity towards breast cancer cells	[126]
*Dendrophthoe falcata*	*Cladosporium perangustum*	AgNPs	30–40	Spherical	Anticancer activity against MCF-7 cell line	[127]
*Commiphora wightii*	*Cladosporium* sp.	AuNPs	5–10	Spherical	Anticancer activity against MCF-7 cell line	[115]
*Pinus densiflora*	*Talaromyces purpureogenus*	AgNPs	25	Round, Triangular	Anticancer activity against A549 cell line	[128]

## Data Availability

No new data were created or analyzed in this study. Data sharing is not applicable to this article.
